# Long- and short-read RNA sequencing from five reproductive organs of boar

**DOI:** 10.1038/s41597-023-02595-0

**Published:** 2023-10-05

**Authors:** Zhipeng Liu, Xia Zhang, Libin Huang, Hailong Huo, Pei Wang, Weizhen Li, Hongmei Dai, Fuhua Yang, Guowen Fu, Guiying Zhao, Yu H. Sun, Jinlong Huo

**Affiliations:** 1 College of Animal Science and Technology, Yunnan Agricultural University, Kunming, 650201 Yunnan China; 2 College of Life Science, Lyuliang University, Lvliang, 033001 Shanxi China; 3 Department of Biology, College of Science, Northeastern University, Boston, Massachusetts 02115 USA; 4 Yunnan Open University, Kunming, 650500 Yunnan China; 5 College of Veterinary Medicine, Yunnan Agricultural University, Kunming, 650201 Yunnan China; 6 Department of Biology, University of Rochester, Rochester, New York 14627 USA

**Keywords:** Sequencing, Animal breeding

## Abstract

The production of semen in boars involves multiple reproductive glands, including the testis (Tes), epididymis (Epi), vesicular gland (VG), prostate gland (PG), and bulbourethral gland (BG). However, previous studies on boar reproduction primarily focused on the testis, with little attention paid to the other glands. Here, we integrated single-molecule long-read sequencing with short-read sequencing to characterize the RNA landscape from five glands of Banna mini-pig inbred line (BMI) and Diannan small-ear pigs (DSE). We identified 110,996 full-length isoforms from 22,298 genes, and classified the alternative splicing (AS) events in these five glands. Transcriptome-wide variation analysis indicated that the number of single nucleotide polymorphisms (SNPs) in five tissues of BMI was significantly lower than that in the non-inbred pig, DSE, revealing the effect of inbreeding on BMI. Additionally, we performed small-RNA sequencing and identified 299 novel miRNAs across all glands. Overall, our findings provide a comprehensive overview of the RNA landscape within these five glands, paving the path for future investigations on reproductive biology and the impact of inbreeding on pig transcriptome.

## Background & Summary

The Banna mini-pig inbred line (BMI) was developed through rigorous selection and continuous inbreeding using the Diannan small-ear (DSE) pigs via a full-sibling or parent-offspring mating strategy^1,2^. Due to its high degree of homozygosity in the genome, BMI serves as a valuable experimental resource for biomedical research and as an organ donor for xenotransplantation^1^. However, after more than 20 generations of inbreeding^2^, the reproductive capacity of boars decreases, thereby limiting the propagation and utilization of BMI. Male fertility requires the coordination of various organs of the male urogenital system, such as the testis, epididymis, and male accessory glands (vesicular gland, prostate gland, and bulbourethral gland), which secrete seminal plasma responsible for regulating capacitation and acrosomal exocytosis^3,4^. Due to the lack of high-quality transcriptomic resource of five pig reproductive organs so far, we aim for a comprehensive understanding of long RNA and miRNA landscape in these organs, with paired samples obtained from both inbred and non-inbred boars.

Single-molecule long-read sequencing (third-generation sequencing; PacBio Iso-seq) technology offers a valuable approach for identifying full-length RNA transcripts^5^. Currently, the PacBio system can sequence full-length transcripts up to 30 kb^6^ but with low depth (about 0.6 million full-length non-chimeric reads per sample in our data) and a high error rate in base calling (~15%)^7^. Illumina paired-end RNA-seq (second-generation sequencing) sequences fragmented RNAs, producing reads with much higher depth (about 24 million paired-end reads per sample in our data) and accuracy^8,9^. The combination of long-read and short-read sequencing maximizes their respective advantages, uncovering isoform complexity and providing quantitative measurements. The strategy combining Iso-seq and RNA-seq techniques has been proven effective for improving pig genome annotation^10^ and identifying intact long RNAs in sperm^6^.

**Fig. 1:**
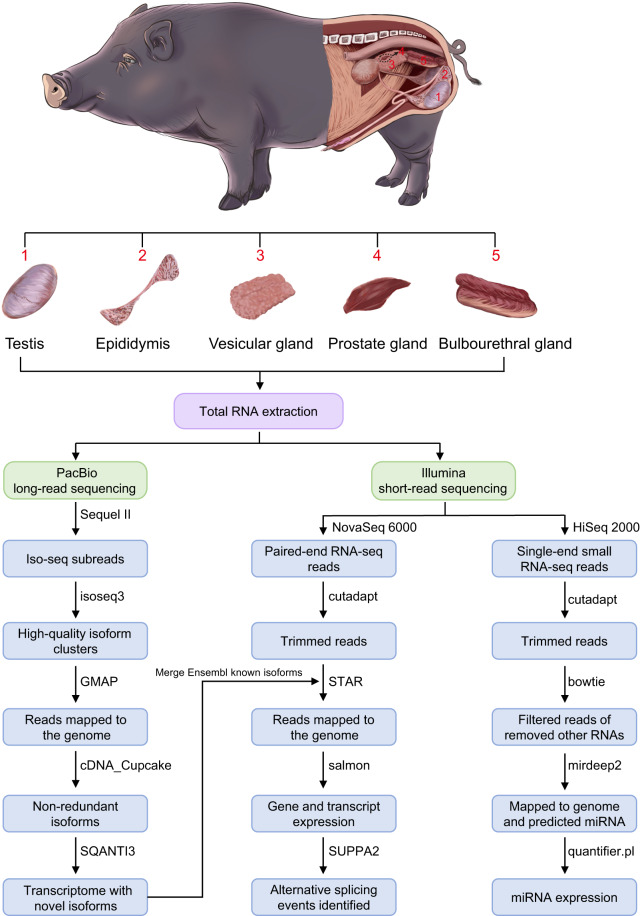
Sample collection and data analysis workflow.

Here, we sequenced five tissues from BMI and DSE using PacBio long-read sequencing, Illumina paired-end RNA sequencing, and Illumina single-end small RNA sequencing. A diagram of the workflow used in this study is presented in Fig. 1. In total, we identified 110,996 full-length transcripts from 22,298 genes, and 299 novel miRNAs across five tissues. To our knowledge, this is the first study to perform long-read sequencing simultaneously on the testis, epididymis, vesicular gland, prostate gland, and bulbourethral gland of pig. This rich resource will benefit further mechanistic studies in reproductive biology, animal science, and translational research.

## Methods

### Samples and sequencing

In this study, we collected tissue samples from the testis, epididymis, vesicular gland, prostate gland, and bulbourethral gland of 12-month-old adult boars, from both BMI and DSE. All animal procedures were approved by Yunnan Agricultural University’s Life Science Ethics Committee and carried out in accordance with the People’s Republic of China’s guidelines for the care and use of laboratory animals (Approval No. 2006-398). The collected samples were promptly snap-frozen in liquid nitrogen and stored at −80 °C. Total RNAs from thirty tissue samples were extracted using TRNzol Universal Reagent (Tiangen, China). The RNA purity was assessed using a NanoDrop One Spectrophotometer (Thermo Fisher Scientific, USA), following accurate RNA quantification performed using a Qubit 3.0 Fluorometer (Invitrogen, USA). Additionally, the RIN value and 28 S/18 S ratio were determined using an Agilent 2100 Bioanalyzer (Agilent Technologies, USA).

For PacBio Iso-seq sequencing, RNA was reverse-transcribed into cDNA using the SMARTer PCR cDNA Synthesis Kit (TakaRa, China). PCR amplification was carried out using the PrimeSTAR GXL DNA polymerase (TakaRa, China). PacBio SMRTbell libraries were constructed using the SMRTbell Template Prep Kit 1.0 (PacBio, USA). Finally, sequencing (Iso-seq) was performed on the Pacific Bioscience Sequel II platform in GrandOmics Biosciences Co., Ltd. (Wuhan, China).

For RNA-seq sequencing, libraries were constructed using the NEBNext Ultra RNA Library Prep Kit for Illumina (NEB, USA), which mainly included mRNA purification with poly-T oligo-attached magnetic beads, fragmentation of mRNA, synthesis of both first- and second-strand cDNA, and PCR amplification. Sequencing was performed on the Illumina NovaSeq 6000 platform in Novogene Co., Ltd. (Beijing, China).

For small RNA sequencing, libraries were constructed using the NEBNext Multiplex Small RNA Library Prep Set for Illumina (NEB, USA), which mainly included ligation of both 3′ SR adaptor and 5′end adapter, synthesis of first-strand cDNA, PCR amplification, purification of products on the polyacrylamide gel, recovery of DNA fragments. Sequencing was performed on Illumina NovaSeq 6000 platform in Novogene Co., Ltd. (Beijing, China).

### PacBio long-read sequencing data analysis

The BAM files of PacBio subreads underwent processing using SMRT Link (v9.0), following the IsoSeq3 (v3.4.0) pipeline. The Iso-seq long-read processing involved circular consensus sequence (CCS) creation using “ccs” with the parameter “--min-rq 0.9”, primer removal and demultiplexing using “lima” with parameters “--isoseq --peek-guess”. Next, to generate full-length non-chimeric (FLNC) reads, we removed chimeric reads and trimmed poly-A tails using the “isoseq refine” with parameter “--require-polya”. To obtain non-redundant isoforms in all samples, we merged FLNC.bam files and ran the clustering step using “isoseq cluster” with the parameter “--use-qvs”. This step generated high-quality (HQ) and low-quality (LQ) HiFi (High Fidelity) isoforms based on the predicted accuracy of the clustered sequences (isoforms). HQ HiFi isoforms have a predicted accuracy ≥ 0.99, while LQ HiFi isoforms have a predicted accuracy < 0.99. Subsequently, the HQ HiFi isoforms, i.e., the draft full-length transcriptome, were aligned to the *Sus scrofa* reference genome assembly Sscrofa11.1 using GMAP (v2018)^11^ with parameters “-B 5 -A -f samse --nofails -n 1”. Finally, cDNA_Cupcake (v29.0.0) was employed to collapse full-length isoforms into the full-length transcriptome assembly, and 5′ degraded isoforms were filtered. We also developed our own program, PacBio_pbIsoCollapse, to further clean up the redundant isoforms and generate the final assembly. In addition, SQANTI3 (v5.0)^12^ was used to estimate the transcriptome assemblies generated from the IsoSeq v3 and cDNA_Cupcake pipelines. The reference annotation (*Sus scrofa* annotation release-107) was used as a reference to compare our assembly with known isoforms. We used SQANTI3 to detect and characterize novel isoforms, including isoforms originating from novel loci, antisense isoforms derived from known genes, and fusion isoforms. Furthermore, FSM (Full Splice Match) and ISM (Incomplete Splice Match) were defined as known isoforms, and the isoforms of NIC (novel in catalog) and NNC (novel not in catalog) were defined as novel transcripts of known genes, and the fusion isoforms, genic isoforms, intergenic isoforms, and antisense isoforms were defined as transcripts of novel genes. We combined these novel transcripts with the known Ensembl transcripts into a merged.gtf file.

To assess the saturation of isoforms, we used cDNA_Cupcake’s “make_file_for_subsampling_from_collapsed.py” script to obtain the number of all full-length sequences from both known and novel isoforms, and then performed rarefaction analysis using “subsample_with_category.py” from the cDNA_Cupcake package. To call SNP, we generated the locus and the number of full-length reads associated with transcript isoforms using “select_loci_to_phase.py” script (referred to https://github.com/Magdoll/cDNA_Cupcake/wiki/IsoPhase:-Haplotyping-using-Iso-Seq-data). Then we created Variant Call Format (VCF) files using modified “run_phasing_in_dir.sh” script, and calculated the SNP density from each vcf file using vcftools (v0.1.16)^13^ with parameter “--SNPdensity 100000”.

To calculate the coding potential of novel isoforms, we employed four different approaches: Coding Potential Calculator v2 (CPC2)^14^, Coding-Potential Assessment Tool v3.0.4 (CPAT)^15^, Pfam database (v35.0)^16^ and GeneMarkS-T (GMST)^17^. All four methods classified RNA sequences as coding or non-coding. CPC2 was mainly used for transcriptome assemblies. CPAT was used to annotate protein coding genes and non-coding genes. For the Pfam, we first translated RNA sequences to protein sequences using ORFfinder (v0.4.3)^18^ with parameters “-ml 200 -outfmt 0”. Then we aligned the protein sequences to the Pfam database using pfam_scan (v1.6)^16^ with default parameters. Moreover, we predicted the coding regions of novel isoforms using iterative self-training and hidden semi-Markov models of the GeneMarkS-T (GMST) algorithm. The union of all “coding” transcripts predicted by these four approaches were defined as “novel protein-coding”. The rest transcripts with >200 bp in length were categorized as “novel lncRNA”, and the remaining transcripts were labeled as “else”.

For the “novel protein-coding” transcripts, the RNA sequences were aligned to the UniProt database (release 2022_05) and the NR (Non-Redundant Protein Sequence) database (https://ftp.ncbi.nih.gov/blast/db/FASTA/nr.gz) using diamond (v2.0.15.153)^19^ “blastx” with the following parameters “--outfmt 6 --long-reads --max-target-seqs 1 --id 50 --more-sensitive”.

### Illumina paired-end sequencing data analysis

RNA-seq short reads were trimmed using cutadapt (v3.0)^20^, removing adapter sequences and low-quality bases. The paired-end short reads were aligned to the pig reference genome Sscrofa11.1 with annotation from merged.gtf using STAR (v2.7.10a)^21^ with parameters “--alignEndsType EndToEnd --outFilterIntronMotifs RemoveNoncanonicalUnannotated --outSAMattributes All --outSAMtype BAM”. The generated alignment bam files were sorted and indexed using samtools (v1.6)^22^. The proportions of reads that fell into genes were calculated using featureCounts (v2.0.1)^23^ with the parameters “-p -t exon -g gene_id -a merged.gtf”. The transcript integrity number (TIN) and the distribution of reads over genomic features were calculated using read_distribution.py and tin.py from the RseQC package (v5.0.1)^24^. The expression of genes and isoforms was quantified using the salmon (v1.9.0)^25^ “quant” module. Principal component analysis (PCA) and sample hierarchical clustering were performed using “prcomp” and “cor” functions from the stats package of R.

### Identification of alternative splicing (AS) and differentially alternative splicing (DAS) events

We detected seven types of AS events using SUPPA2 (v2.3)^26^, including skipping exon (SE), alternative 5′ splice sites (A5), alternative 3′ splice sites (A3), retained intron (RI), mutually exclusive exon (MX), alternative first exon (AF), and alternative last exon (AL). We employed SUPPA2 to convert annotation files containing genomic coordinates and transcript isoforms into possible alternative splicing events, and then applied SUPPA2 “generateEvent” with the parameter “-f ioe” to generate the ioe file containing alternative splicing events from the merged.gtf. Further, SUPPA2 “psiPerEvent” was executed to generate the PSI (percent spliced in) expression level of each alternative splicing event. Finally, to identify DAS events between five different tissues from BMI and DSE, we compared the PSI values of alternative splicing events by breed and tissue using the SUPPA2 “diffSplice”.

### Illumina small RNA sequencing analysis

For small RNA analysis, The 3′ adapter and low-quality sequences were first removed using cutadapt (v3.0)^20^. GC content and length of reads were calculated using seqkit (v2.1.0)^27^ with parameters “fx2tab -g -l”. The rRNAs, tRNA, snRNAs, and snoRNAs were filtered using bowtie (v1.3.1)^28^. The novel miRNAs were further predicted using miRDeep2 (v0.1.3)^29^, and the known miRNAs and novel miRNAs were quantified using quantifier.pl from miRDeep2.

## Data Records

The sequencing data were deposited in the NCBI Gene Expression Omnibus under the accession number GSE230506^30^. The full-length transcriptome assembly annotation file^31^, full-length transcriptome sequences^32^, novel coding isoforms annotation^33^, splice data^34^, quantification of gene and miRNA expression and predicted novel miRNA sequences^35^ have been deposited in the Figshare database.

## Technical Validation

### High-quality data from long-read sequencing

**Fig. 2:**
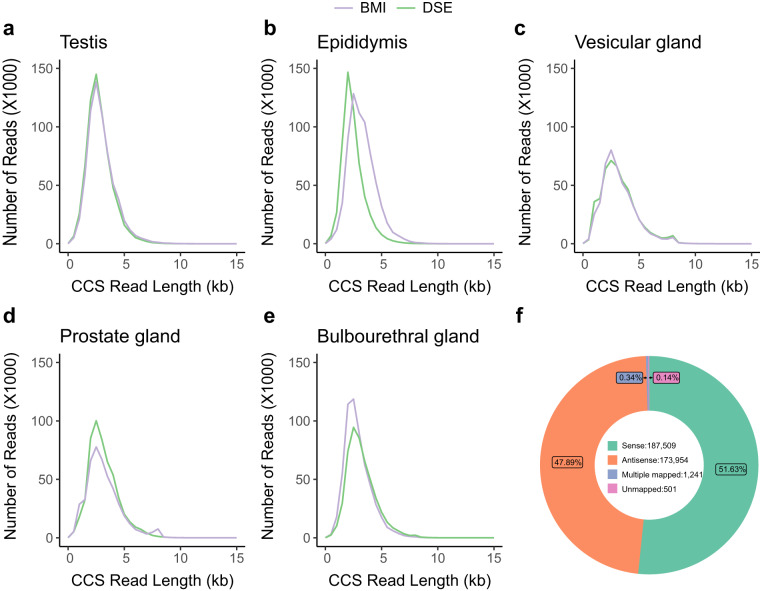
Length distribution of circular consensus sequences (CCS) in (**a**) testis, (**b**) epididymis, (**c**) vesicular gland, (**d**) prostate gland, and (**e**) bulbourethral gland from BMI (Banna mini-pig inbred line) and DSE (Diannan small-ear pig). (**f**) Statistics of HQ HiFi reads mapped to the genome in all samples.

**Fig. 3:**
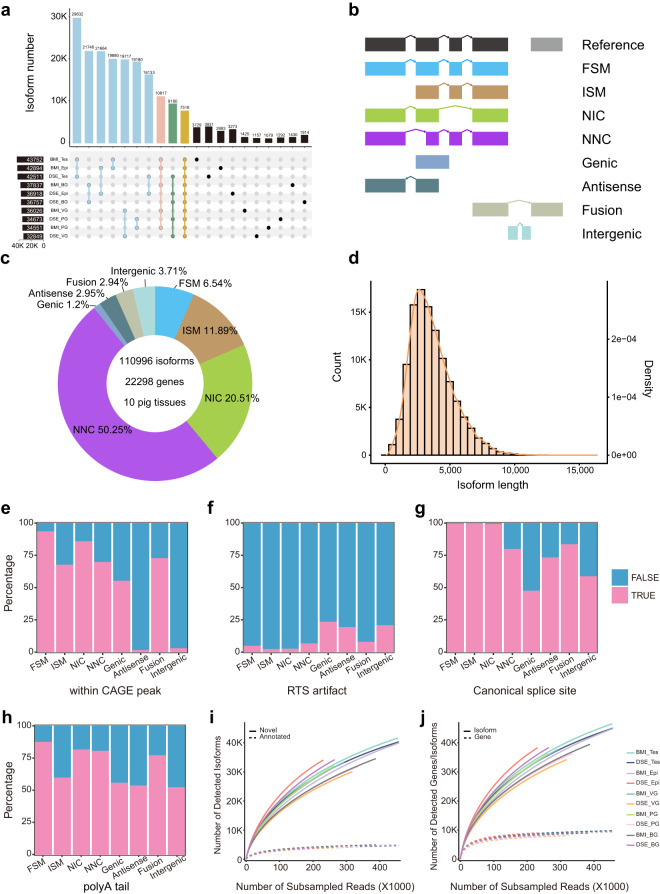
Landscape of long-read transcriptome in ten samples. (**a**) UpSet plot showing the distribution of tissue-specific and shared isoforms across all samples. (**b**) Diagram of isoforms classified by SQANTI3. (**c**) Percentage of identified isoform types. (**d**) Length distribution and density curve of all non-redundant isoforms. The density was calculated by density function of R. (**e**-**h**) Quality features of full-length isoforms provided by SQANTI3, including CAGE peak support (**e**), reverse transcriptase template switching (RTS) (**f**), canonical splice site (**g**), and poly-A tail (**h**). (**i**) Rarefaction plot of novel isoforms and annotated isoforms. (**j**) Rarefaction plot of genes and isoforms. FSM, full splice match; ISM, incomplete splice match; NIC, novel in catalog; NNC, novel not in catalog. Tes, testis; Epi, epididymis; VG, vesicular gland; PG, prostate gland; BG, bulbourethral gland.

For each sample, we generated ~50 Gb (gigabases) of PacBio subreads. The number of CCS, FLNC reads, total HiFi isoforms, and the mapping ratio of HQ HiFi isoforms for each sample have been provided in Table S1. The length distribution of CCS ranged from 2–3 kb across all five tissues, including testis, epididymis, vesicular gland, prostate gland, and bulbourethral gland (Fig. 2a–e). We mapped the HQ HiFi isoforms with more than 99% accuracy to the pig genome (Sscrofa11.1) using GMAP, with the mapping rate exceeding 99% (Table S1, Fig. 2f). After removing redundant sequences and 5′ degraded products using cDNA_Cupcake and PacBio_pbIsoCollapse, we identified an average of 37,876 non-redundant full-length isoforms per sample. Notably, the DSE testis exhibited the highest number of sample-specific isoforms, with 3,931 isoforms identified (Fig. 3a).

We identified 110,996 non-redundant isoforms in BMI and DSE, and classified them into eight types based on the Ensembl 107 *Sus scrofa* reference annotation. Of these, 6.54% (7,260) were full splice match (FSM) isoforms that match completely to known transcripts; 11.89% (13,201) were incomplete splice match (ISM), corresponding to isoforms matching a subset of exons of known transcripts; 20.51% (22,765) and 50.25% (55,774) were from the novel in catalog (NIC) and novel not in catalog (NNC) of known genes, respectively; 1.2% (1,334), 2.95% (3,276), 2.94% (3,267), and 3.71% (4,119) were Genic, Antisense, Fusion and Intergenic novel isoforms, respectively (Fig. 3b,c). The length of these isoforms was distributed between 1,000–10,000 bp, with the highest density around 3,000 bp (Fig. 3d). The classification results for each sample are listed in Table S2. In addition, we evaluated the reliability of the full-length isoforms using a variety of quality metrics from SQANTI3, including the overlap of 5′ transcript ends with Ensembl 107 *Sus scrofa* reference annotation (see refTSS deposited at Figshare^36^), reverse transcriptase template switching (RTS), canonical splice site, and 3′ ends with polyA tails (Fig. 3e–h). Compared with FSM, NIC and NNC, ISM had a lower ratio of 5′-end overlapping with Ensembl transcripts, which may be because some ISM contained partial reverse transcription or mRNA degradation products. As a result, ISMs were removed when we merged the PacBio transcriptome into the Ensembl known transcriptome. Furthermore, we found that the novel isoforms (genic, antisense, and intergenic) from novel genes exhibited a higher proportion of the RTS artifacts (Fig. 3f). Rarefaction curve analysis revealed that saturation occurred on known isoforms but not on novel isoforms (Fig. 3i), and on the gene level but not on the isoform level (Fig. 3j), implying that the increase in novel isoforms is more likely attributed to known genes rather than novel genes.

### Characteristics of full-length isoforms

**Fig. 4:**
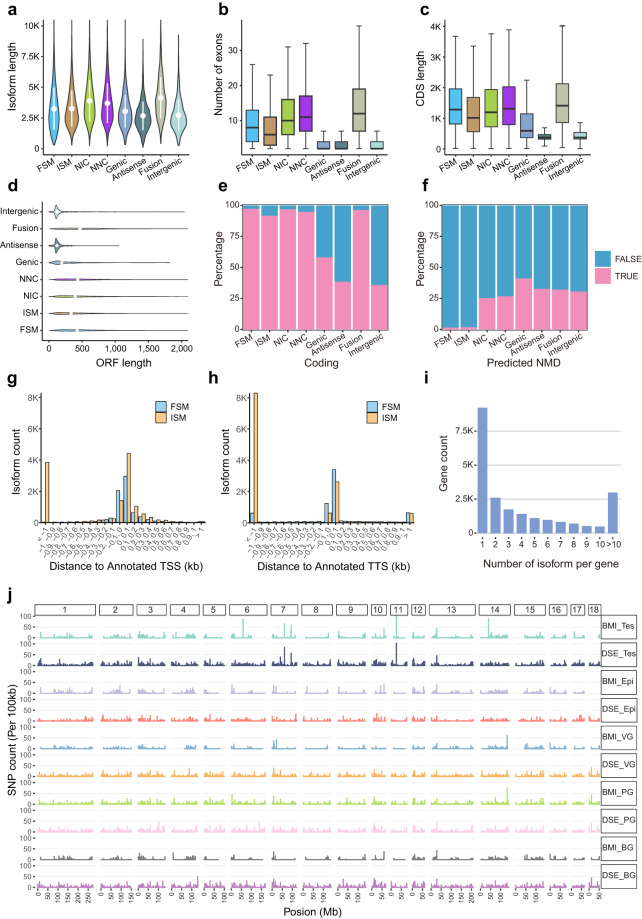
Characteristics of identified isoforms. Plots showing the length of isoforms (**a**), the number of exons (**b**), the length of coding sequence (**c**), the length of ORF (**d**), the proportion of protein-coding genes(**e**), and the proportion of predicted NMD (**f**) in known isoforms (FSM, ISM) and novel isoforms (NIC, NNC, Genic, Antisense, Fusion, Intergenic). Histogram showing the distance to annotated transcription start site (TSS) (**g**) and transcription termination site (TTS) (**h**) for FSM and ISM. (**i**) Number of isoforms per gene. (**j)** Single-nucleotide polymorphism (SNP) count using 100 kb of sequencing length as window size in the ten samples. Note: NMD, nonsense-mediated mRNA decay; FSM, full splice match; ISM, incomplete splice match; NIC, novel in catalog; NNC, novel not in catalog. Tes, testis; Epi, epididymis; VG, vesicular gland; PG, prostate gland; BG, bulbourethral gland.

We then characterized the full-length isoforms in the eight categories mentioned above. Compared with the other seven categories, fusion isoforms had longer transcript lengths, more exons, and longer coding sequences (Fig. 4a–c). The Intergenic and Antisense isoforms exhibited shorter ORF lengths than the other classes (Fig. 4d), signifying a lower abundance of coding transcripts in these two categories (Fig. 4e). Moreover, novel isoforms manifested more nonsense-mediated mRNA decay (NMD) than known isoforms (Fig. 4f). Comparison with FSM, more ISM isoforms fell into downstream of 5′ start end of reference transcripts (Fig. 4g) and upstream of 3′ termination end of reference transcripts (Fig. 4h). We also calculated the number of isoforms for per gene, identifying 13,088 genes with multiple isoforms and 2,964 genes with more than ten isoforms (Fig. 4i). Furthermore, we analyzed SNPs in these five tissues and observed a significantly lower number of SNPs in BMI compared to DSE (Fig. 4j). Specifically, in testis, the average log_2_ SNP count of BMI was 0.079, while that of DSE was 0.146 (*p* < 2.2 × 10^−16^); in the epididymis, the average log_2_ SNP count of BMI and DSE were 0.071 and 0.085 respectively (*p* = 7.387 × 10^−4^); in the vesicular gland, the average log_2_ SNP count of BMI and DSE were 0.048 and 0.112, respectively (*p* < 2.2e × 10^−16^); in the prostate gland, the average log_2_ SNP count of BMI and DSE were 0.099 and 0.139, respectively (*p* = 4.373 × 10^−16^); and in the bulbourethral gland, the average log_2_ SNP count of BMI and DSE were 0.055 and 0.118, respectively (*p* < 2.2 × 10^−16^), implying the effect of inbreeding on homozygosity of the pig genome.

**Fig. 5:**
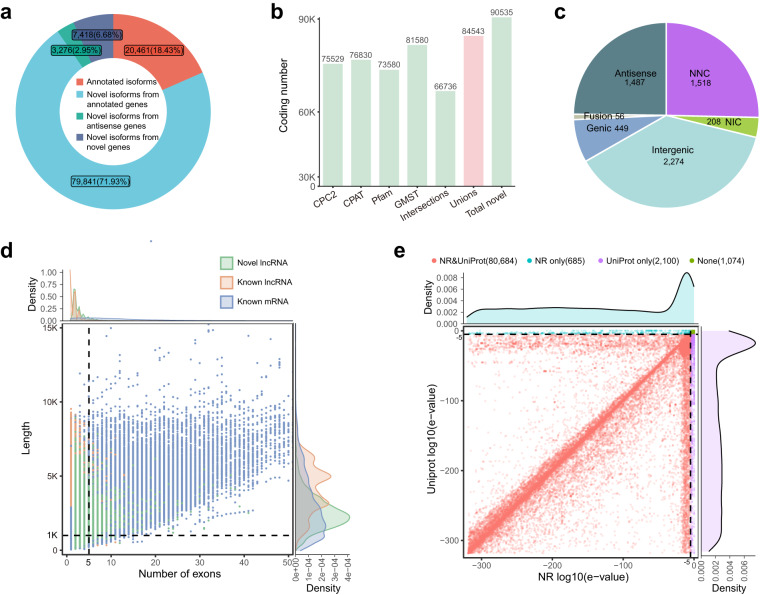
Analysis of novel coding isoforms and novel non-coding isoforms. (**a**) Percentage of isoforms in each annotation category. (**b**) Number of coding isoforms predicted by four approaches: CPC2, CPAT, GMST and Pfam. The pink bar indicates the union of predicted coding isoform from four approaches. (**c**) The number of lncRNA in each category. (**d**) The scatter plot shows the number of exons (x axis) and length distribution (y axis) of novel lncRNA, known lncRNA and known mRNA. Green dot: novel lncRNA; Orange dot: known lncRNA; Blue-gray: known mRNA. The vertical dashed line represents the 5 exons. The horizontal dashed line represents the 1 kb transcript length (**e**) Sequence annotation analysis for novel coding isoforms. The red points represent significance (log10(e-value) <−5) according to both NR (Non-Redundant protein sequence database) and Uniprot (Universal protein database). Blue points indicate NR only, purple points indicate UniProt only, and green points indicate no significance in either analysis.

We predicted the coding potential of a total of 90,535 novel isoforms using CPC2, CPAT, Pfam, and GMST, including those from annotated genes, antisense genes, and novel genes (Fig. 5a), of which 84,543 were protein-coding isoforms. The results indicated that most lncRNA were intergenic isoforms, whose exon distribution was similar to that of known lncRNAs (Fig. 5b–d). To further investigate these novel isoforms with protein-coding potential, we performed functional annotation using the UniProt database and the NR (Non-Redundant Protein Sequence) database. The result shows that most of the predicted peptides aligned confidently to both databases with significant e-values, suggesting their ability to encode functional proteins (Fig. 5e).

### Analysis of paired-ends sequence data

**Fig. 6:**
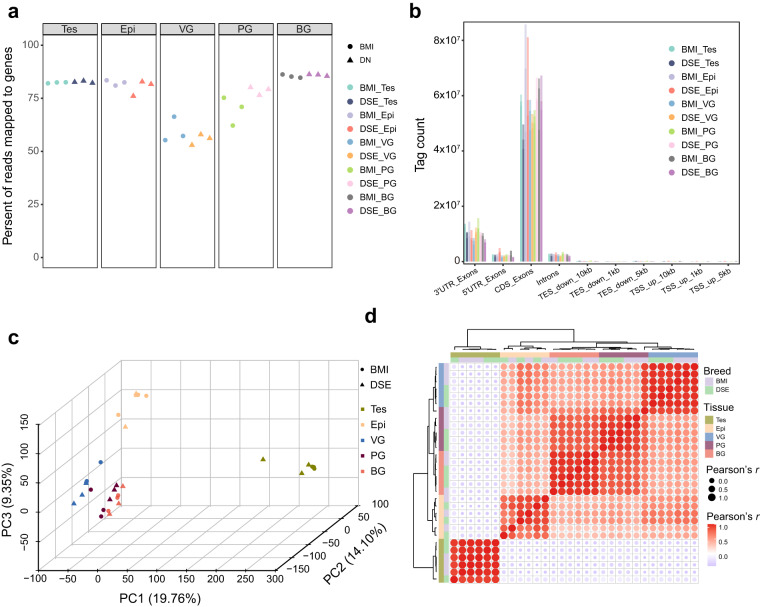
Analysis of short-read sequencing data. (**a**) The proportion of reads mapped to genes. (**b**) The number of reads mapped to various gene regions. (**c**) Principal component analysis (PCA) of all samples. (**d**) Correlation analysis of all samples. Tes, testis; Epi, epididymis; VG, vesicular gland; PG, prostate gland; BG, bulbourethral gland.

A total of 230 Gb of clean reads were generated using the NovaSeq 6000 platform. The statistics of clean reads, clean reads base, Q30, GC content, mapped reads, and TIN (Transcript Integrity Number) statistics are shown in Table S3. The median TIN scores ranged from 67.68 to 81.33, and the mean was 77.88 (Table S3). More than 55% of reads fell into genes (Fig. 6a), with most reads mapped to exonic regions, while fewer reads mapped to intronic regions (Fig. 6b), indicating high quality RNA-seq data obtained from five porcine tissues.

Principal component analysis (PCA) indicated that the first and second principal components (PCs, 19.76% and 14.10% explained variance, respectively) significantly distinguished testis, epididymis, and other glands, and the third PC (9.35% explained variance) further separated the VG, PG, and BG accessory glands (Fig. 6c). The correlation values between the same tissues were greater than 0.95, whereas those between different tissues were less than 0.5 (Fig. 6d).

### Characterization of AS and DAS events

**Fig. 7:**
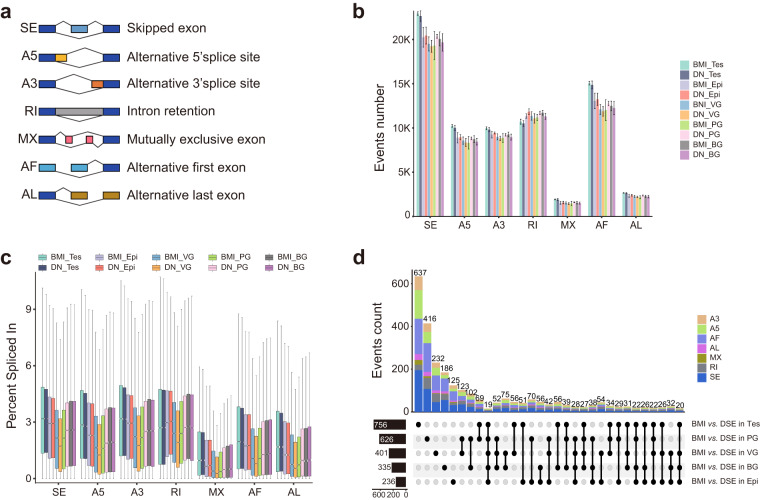
Identification of AS (Alternative Splicing) and DAS (Differential Alternative Splicing). (**a**) Diagram of seven types of AS events. (**b**) Number of AS events in thirty samples. (**c**) Percentage of alternative splicing (AS) events across different samples. (**d**) Differential alternative splicing between BMI and DSE in five tissues. Tes, testis; Epi, epididymis; VG, vesicular gland; PG, prostate gland; BG, bulbourethral gland.

To gain insight into the AS patterns of full-length isoforms across five organs and two breeds, we counted the number of SE, A5, A3, RI, MX, AF, and AL types, respectively (Fig. 7a). Of note, SE was the most prevalent type of AS across all glands (Fig. 7b), and the PSI of AS in testes was significantly higher than that in other glands (Fig. 7c). Additionally, DAS analysis in five tissues of BMI and DSE indicated that total DAS and specific DAS were also higher in the testes than in the other glands (Fig. 7d).

### Analysis of small RNA sequencing data

**Fig. 8:**
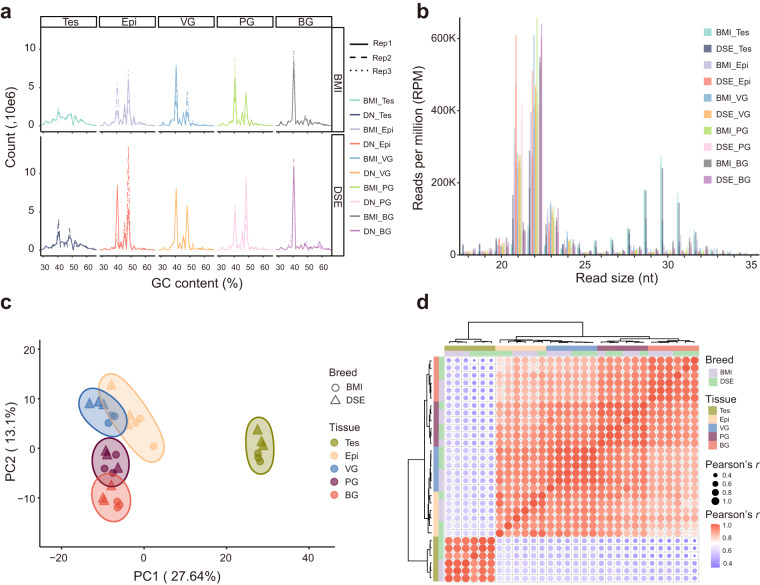
Analysis of small RNA data. (**a**) GC content of small RNA reads in thirty samples. (**b**) Distribution of small RNA length in thirty samples. (**c**) Principal component analysis (PCA) of miRNA abundance in thirty samples. (**d**) Correlation analysis of small (micro) RNA abundance in thirty samples.

Small RNA analysis of the five tissues indicated a higher consistency of GC content within the same gland (Fig. 8a, Table S4). Notably, two small RNA peaks displayed at 21–23 nt and 28–31 nt in the testes respectively, indicating the presence of both miRNA and piRNA in the testes, which was consistent with the previous studies^37^. For the remaining four tissues, only one peak was observed at 21–23 nt, corresponding to miRNAs (Fig. 8b). PCA analysis of miRNAs further confirmed a clear separation of the testes from the other four glands on PC1 (27.64% variation), and the separation of epididymis from the vesicular gland, prostate gland, bulbourethral gland on PC2 (13.1% variation) (Fig. 8c). Correlation analysis revealed that testes miRNA expressions were much less correlated with the other four glands, whereas the epididymis, vesicular glands, prostate, and bulbourethral glands exhibited higher correlation with each other (Fig. 8d).

## Usage Notes

The Iso-seq data enables accurate measurements of full-length transcripts and alternative splicing events across five reproductive glands in two pig breeds. Complementing this, the paired-end RNA-seq data can be used to quantify gene expression and assess alternative isoform usage. The small RNA-seq data facilitates the exploration of multiple small non-coding RNAs, including miRNA, piRNA, snRNA, snoRNA, and tRNA. The miRNA expression data will be useful for understanding the miRNA-mRNA regulatory networks in five tissues, and identifying novel miRNAs.

The present study provides a comprehensive resource of five BMI and DSE reproductive glands. As the dataset contains both long-read and short-read sequencing data, this rich sequencing dataset paves the avenue for quantifying gene or transcript expression, evaluating alternative splicing, delving novel transcripts, and improving the annotation of the pig genome.

### Supplementary information

SUPPLEMENTARY INFORMATION

## Data Availability

All software used in this study is open access and parameters were clearly described in the Methods section. If no detailed parameters of the software are mentioned, the default parameters suggested by the developer were applied. A comprehensive list of software used in this study is provided in this section as well. SMRT Link (v9.0) was used to trim reads of Iso-seq data: https://www.pacb.com/support/software-downloads IsoSeq3 (v3.4.0) was used to cluster isoforms of trimmed reads for Iso-seq data: https://github.com/PacificBiosciences/IsoSeq cDNA_Cupcake (v29.0.0) was used to collapse redundant isoforms: https://github.com/Magdoll/cDNA_Cupcake PacBio_pbIsoCollapse was developed by this study to further remove redundant isoforms: https://github.com/lhuang3s/PacBio_pbIsoCollapse GMAP (v2018-07-04) was used to align the full-length isoforms of Iso-seq to the *Sus scrofa* reference genome: https://bioconda.github.io/recipes/gmap/README.html SQANTI3 (v5.0) was used to compare the Iso-seq assembly transcriptome with the Ensembl transcriptome: https://github.com/ConesaLab/SQANTI3 vcftools (v0.1.16) was used to calculate the density of SNP: https://vcftools.sourceforge.net ORFfinder (v0.4.3) was used to translate RNA sequences to protein sequences: https://ftp.ncbi.nlm.nih.gov/genomes/TOOLS/ORFfinder pfam_scan (v1.6) was used to search domain against a library of Pfam HMMs: https://bioconda.github.io/recipes/pfam_scan/README.html CPC2 and CPAT (v3.0.4) were used to evaluate the coding potential of novel isoforms: http://cpc2.gao-lab.org; https://cpat.readthedocs.io/en/latest diamond (v2.0.15.153) was used to align the novel isoforms’ sequence to NR and UniProt: https://github.com/bbuchfink/diamond cutadapt (v3.0) was used to trim reads of paired-end RNA-seq and single-end small RNA-seq data: https://cutadapt.readthedocs.io/en/stable STAR (v2.7.10a) was used to map paired-end sequence reads to the *Sus scrofa* reference genome: https://github.com/alexdobin/STAR SAMtools (v1.6) was used to sort and build an index for short-reads align BAM files: https://github.com/samtools/samtools featureCounts (v2.0.1) was used to calculate the gene counts: https://subread.sourceforge.net/featureCounts.html RseQC (v5.0.1) was used to evaluate the sequence quality: https://rseqc.sourceforge.net salmon (v1.9.0) was used to calculate the gene expression and transcript expression: https://combine-lab.github.io/salmon SUPPA2 (v2.3) was used to analyze alternative splicing and differential alternative splicing: https://github.com/comprna/SUPPA seqkit (v2.1.0) was used to calculate the GC content of small RNA-seq data: https://github.com/shenwei356/seqkit bowtie (v1.3.1) was used to align miRNA reads to pig genome: https://bowtie-bio.sourceforge.net/index.shtml miRDeep2 (v0.1.3) was used to predict potential miRNA: https://github.com/rajewsky-lab/mirdeep2 scatterplot3d (v0.3-42) was used to plot the 3D PCA map: https://cran.r-project.org/web/packages/scatterplot3d/index.html Other maps were plotted by ggplot2 (v3.4.0): https://ggplot2.tidyverse.org Custom scripts to handle genomic annotations: https://github.com/sunyumail93/Bed12Processing The pipeline code for Iso-seq: https://github.com/zhipengliu92/PipIsoseq The pipeline code for RNA-seq: https://github.com/sunyumail93/PipeRNAseq The pipeline code for small RNA-seq: https://github.com/sunyumail93/PipeSmRNAseq

## References

[1] Huo JL (2022). Genome-wide single nucleotide polymorphism array and whole-genome sequencing reveal the inbreeding progression of Banna minipig inbred line. Anim Genet.

[2] Wang P (2023). Transcriptomic analysis of testis and epididymis tissues from Banna mini-pig inbred line boars with single-molecule long-read sequencing. †. Biol Reprod.

[3] Verze P, Cai T, Lorenzetti S (2016). The role of the prostate in male fertility, health and disease. Nat Rev Urol.

[4] Pavaneli, A. P. P. *et al*. The presence of seminal plasma during liquid storage of pig spermatozoa at 17 °C modulates their ability to elicit *in vitro* capacitation and trigger acrosomal exocytosis. *Int J Mol Sci* **21**, 10.3390/ijms21124520 (2020). 10.3390/ijms21124520 PMC7350249 32630462

[5] Rhoads A, Au KF (2015). PacBio sequencing and its applications. Genomics Proteomics Bioinformatics.

[6] Sun YH (2021). Single-molecule long-read sequencing reveals a conserved intact long RNA profile in sperm. Nat Commun.

[7] Weirather JL (2017). Comprehensive comparison of Pacific Biosciences and Oxford Nanopore Technologies and their applications to transcriptome analysis. F1000Res.

[8] Au KF, Jiang H, Lin L, Xing Y, Wong WH (2010). Detection of splice junctions from paired-end RNA-seq data by SpliceMap. Nucleic Acids Res.

[9] Roberts A, Pachter L (2011). RNA-Seq and find: entering the RNA deep field. Genome Med.

[10] Beiki H (2019). Improved annotation of the domestic pig genome through integration of Iso-Seq and RNA-seq data. BMC Genomics.

[11] Wu TD, Watanabe CK (2005). GMAP: a genomic mapping and alignment program for mRNA and EST sequences. Bioinformatics.

[12] Tardaguila M (2018). SQANTI: extensive characterization of long-read transcript sequences for quality control in full-length transcriptome identification and quantification. Genome Res.

[13] Danecek P (2011). The variant call format and VCFtools. Bioinformatics.

[14] Kang YJ (2017). CPC2: a fast and accurate coding potential calculator based on sequence intrinsic features. Nucleic Acids Res.

[15] Wang L (2013). CPAT: Coding-Potential Assessment Tool using an alignment-free logistic regression model. Nucleic Acids Res.

[16] Tang S, Lomsadze A, Borodovsky M (2015). Identification of protein coding regions in RNA transcripts. Nucleic Acids Res.

[17] Mistry J (2021). Pfam: The protein families database in 2021. Nucleic Acids Res.

[18] Sayers EW (2011). Database resources of the National Center for Biotechnology Information. Nucleic Acids Res.

[19] Buchfink B, Reuter K, Drost HG (2021). Sensitive protein alignments at tree-of-life scale using DIAMOND. Nat Methods.

[20] Martin M (2011). Cutadapt removes adapter sequences from high-throughput sequencing reads. EMBnet J.

[21] Dobin A (2013). STAR: ultrafast universal RNA-seq aligner. Bioinformatics.

[22] Danecek, P. *et al*. Twelve years of SAMtools and BCFtools. *Gigascience* **10**, 10.1093/gigascience/giab008 (2021). 10.1093/gigascience/giab008 PMC7931819 33590861

[23] Liao Y, Smyth GK, Shi W (2014). featureCounts: an efficient general purpose program for assigning sequence reads to genomic features. Bioinformatics.

[24] Wang L, Wang S, Li W (2012). RSeQC: quality control of RNA-seq experiments. Bioinformatics.

[25] Patro R, Duggal G, Love MI, Irizarry RA, Kingsford C (2017). Salmon provides fast and bias-aware quantification of transcript expression. Nat Methods.

[26] Trincado JL (2018). SUPPA2: fast, accurate, and uncertainty-aware differential splicing analysis across multiple conditions. Genome Biol.

[27] Shen W, Le S, Li Y, Hu F (2016). SeqKit: a cross-platform and ultrafast toolkit for FASTA/Q file manipulation. PLoS One.

[28] Langmead B, Trapnell C, Pop M, Salzberg SL (2009). Ultrafast and memory-efficient alignment of short DNA sequences to the human genome. Genome Biol.

[29] An J, Lai J, Lehman ML, Nelson CC (2013). miRDeep*: an integrated application tool for miRNA identification from RNA sequencing data. Nucleic Acids Res.

[30] Liu Z (2023). Gene Expression Omnibus.

[31] Liu Z (2023). figshare.

[32] Liu Z (2023). figshare.

[33] Liu Z (2023). figshare.

[34] Liu Z (2023). figshare.

[35] Liu Z (2023). figshare.

[36] Liu Z (2023). figshare.

[37] Ma X (2022). The piRNAs present in the developing testes of Chinese indigenous Xiang pigs. Theriogenology.

