# Supplementary material for: Long- and short-read RNA sequencing from five reproductive organs of boar
Source: Sci Data. 2023 Oct 5;10:678. doi: 10.1038/s41597-023-02595-0 (PMC10556096; doi:10.1038/s41597-023-02595-0)
Supplement: Supplementary file 1 — SUPPLEMENTARY INFORMATION [file 41597_2023_2595_MOESM1_ESM.docx]

**Long- and short-read RNA sequencing from five reproductive organs of boar**

**Zhipeng Liu^1,7^, Xia Zhang^1,2,7^, Libin Huang^3^, Hailong Huo^4^, Pei Wang^1^, Weizhen Li^5^, Hongmei Dai^1^, Fuhua Yang^1^, Guowen Fu^5^, Guiying Zhao^1^**^✉^**, Yu H. Sun^6^**^✉^ **& Jinlong Huo^1,6^**^✉^

1. College of Animal Science and Technology, Yunnan Agricultural University, Kunming 650201, Yunnan, China

2. College of Life Science, Lyuliang University, Lvliang 033001, Shanxi, China

3. Department of Biology, College of Science, Northeastern University, Boston,Massachusetts 02115, USA

4. Yunnan Open University, Kunming 650500, Yunnan, China

5. College of Veterinary Medicine, Yunnan Agricultural University, Kunming 650201, Yunnan, China

6. Department of Biology, University of Rochester, Rochester, New York 14627, USA

7. These authors contributed equally: Zhipeng Liu, Xia Zhang.

^✉^corresponding author(s): Guiying Zhao (e-mail: zhaoguiying2006@163.com), Yu H. Sun (e-mail: yu.sun.rochester@gmail.com) and Jinlong Huo (e-mail: jinlonghuo973@163.com)

**Table of Contents**

**Table S1** Summary of IsoSeq v3 workflow output

**Table S2** Statistics of Iso-seq transcriptome from five tissues across two breeds

**Table S3** Quality control of RNA-seq data

**Table S4** Quality control of small RNA-seq data

**Table S1** Summary of IsoSeq v3 workflow output

| sample | Generated CCS | Primer removed | FLNC reads | Total HiFi isoforms | HQ HiFi isoforms | High-quality mapped | mapped to "+" | mapped to "-" | Multiple mapped |
| --- | --- | --- | --- | --- | --- | --- | --- | --- | --- |
| BMI_Tes | 667,383 | 665,465 | 663,353 | 53,449 | 53,273 | 53,240 (99.94%) | 27,698 | 25,542 | 106 |
| DSE_Tes | 675,159 | 673,542 | 672,169 | 54,927 | 54,758 | 54,698 (99.89%) | 28,627 | 26,071 | 108 |
| BMI_Epi | 688,891 | 686,771 | 683,914 | 50,802 | 50,642 | 50,605 (99.93%) | 26,650 | 23,955 | 175 |
| DSE_Epi | 546,844 | 545,314 | 541,554 | 53,327 | 53,168 | 53,117 (99.90%) | 28,062 | 25,055 | 295 |
| BMI_VG | 471,177 | 469,846 | 461,518 | 37,623 | 37,503 | 37,466 (99.90%) | 19,238 | 18,228 | 150 |
| DSE_VG | 482,428 | 481,064 | 475,894 | 36,684 | 36,538 | 36,504 (99.91%) | 18,826 | 17,678 | 111 |
| BMI_PG | 461,580 | 460,248 | 457,298 | 36,459 | 36,327 | 36,283 (99.88%) | 18,545 | 17,738 | 191 |
| DSE_PG | 536,779 | 535,458 | 532,033 | 43,235 | 43,062 | 43,035 (99.94%) | 22,054 | 20,981 | 140 |
| BMI_BG | 574,621 | 573,116 | 571,524 | 43,135 | 42,992 | 42,955 (99.91%) | 22,323 | 20,632 | 133 |
| DSE_BG | 503,100 | 502,744 | 499,901 | 43,763 | 43,763 | 43,741 (99.95%) | 22,624 | 21,117 | 253 |

**Table S2** Statistics of Iso-seq transcriptome from five tissues across two breeds

| type | BMI_Tes | DSE_Tes | BMI_Epi | DSE_Epi | BMI_VG | DSE_VG | BMI_PG | DSE_PG | BMI_BG | DSE_BG |
| --- | --- | --- | --- | --- | --- | --- | --- | --- | --- | --- |
| Unique Genes | 13,879 | 13,692 | 13,632 | 13,261 | 12,142 | 11,489 | 12,178 | 11,865 | 12,746 | 12,403 |
| Annotated genes | 11,822 (85.18) | 11,758 (85.87) | 11,650 (85.46) | 11,173 (84.25) | 10,590 (87.22) | 10,113 (88.02) | 10,490 (86.14) | 10,485 (88.37) | 11,384 (89.31) | 10,527 (84.87) |
| Novel genes | 2,057 (14.82) | 1,934 (14.13) | 1,982 (14.54) | 2,088 (15.75) | 1,552 (12.78) | 1,376 (11.98) | 1,688 (13.86) | 1,380 (11.63) | 1,362 (10.69) | 1,876 (15.13) |
| Genes with 1 isoform | 5,787 (41.7) | 5,915 (43.2) | 5,466 (40.1) | 5,763 (43.46) | 5,028 (41.41) | 5,046 (43.92) | 5,343 (43.87) | 4,953 (41.74) | 5,012 (39.32) | 5,237 (42.22) |
| Genes with >10 isoforms | 564 (4.06) | 546 (3.99) | 490 (3.59) | 286 (2.16) | 356 (2.93) | 321 (2.79) | 341 (2.8) | 314 (2.65) | 318 (2.49) | 384 (3.1) |
| Genes with 2-10 isoforms | 7,528 (54.24) | 7,231 (52.81) | 7,676 (56.31) | 7,212 (54.39) | 6,758 (55.66) | 6,122 (53.29) | 6,494 (53.33) | 6,598 (55.61) | 7,416 (58.18) | 6,782 (54.68) |
| Isoforms | 43,752 | 42,511 | 42,894 | 36,918 | 36,026 | 32,849 | 34,551 | 34,673 | 37,837 | 36,757 |
| Annotated isoforms | 9,443 (21.58) | 9,221 (21.69) | 10,976 (25.59) | 10,650 (28.85) | 9,927 (27.56) | 9390 (28.59) | 9,812 (28.4) | 10,326 (29.78) | 10,962 (28.97) | 10,488 (28.53) |
| Novel isoforms from annotated genes | 31,191 (71.29) | 30,311 (71.3) | 29,020 (67.66) | 23,537 (63.75) | 23,909 (66.37) | 21,531 (65.55) | 22,474 (65.05) | 22,388 (64.57) | 24,925 (65.87) | 23,746 (64.6) |
| Novel isoforms from antisense genes | 839 (1.92) | 787 (1.85) | 829 (1.93) | 803 (2.18) | 575 (1.6) | 517 (1.57) | 668 (1.93) | 487 (1.4) | 493 (1.3) | 739 (2.01) |
| Novel isoforms from novel genes | 2,279 (5.21) | 2,192 (5.16) | 2,069 (4.82) | 1,928 (5.22) | 1,615 (4.48) | 1,411 (4.3) | 1,597 (4.62) | 1,472 (4.25) | 1,457 (3.85) | 1,784 (4.85) |
| Coding isoforms | 41,573 (95.02) | 40,496 (95.26) | 40,191 (93.7) | 33,951 (91.96) | 33,765 (93.72) | 30,634 (93.26) | 32,009 (92.64) | 32,441 (93.56) | 35,740 (94.46) | 33,888 (92.19) |
| Non-coding isoforms | 2,179 (4.98) | 2,015 (4.74) | 2,703 (6.3) | 2,967 (8.04) | 2261 (6.28) | 2,215 (6.74) | 2,542 (7.36) | 2,232 (6.44) | 2,097 (5.54) | 2,869 (7.81) |
| FSM | 4,840 (11.06) | 4,680 (11.01) | 4,920 (11.47) | 4,211 (11.41) | 4,379 (12.16) | 4,144 (12.62) | 4,208 (12.18) | 4,432 (12.78) | 4,903 (12.96) | 4,178 (11.37) |
| Fusion | 1,322 (3.02) | 1,278 (3.01) | 1,195 (2.79) | 898 (2.43) | 948 (2.63) | 817 (2.49) | 826 (2.39) | 853 (2.46) | 935 (2.47) | 894 (2.43) |
| Genic | 349 (0.8) | 318 (0.75) | 372 (0.87) | 319 (0.86) | 304 (0.84) | 243 (0.74) | 321 (0.93) | 263 (0.76) | 281 (0.74) | 388 (1.06) |
| Intergenic | 952 (2.18) | 909 (2.14) | 864 (2.01) | 1,019 (2.76) | 662 (1.84) | 589 (1.79) | 767 (2.22) | 616 (1.78) | 513 (1.36) | 881 (2.4) |
| ISM | 4,603 (10.52) | 4,541 (10.68) | 6,056 (14.12) | 6,439 (17.44) | 5,548 (15.4) | 5,246 (15.97) | 5,604 (16.22) | 5,894 (17) | 6,059 (16.01) | 6,310 (17.17) |
| NIC | 8,683 (19.85) | 8,247 (19.4) | 10,740 (25.04) | 9,692 (26.25) | 9,502 (26.38) | 8,531 (25.97) | 8,941 (25.88) | 8,749 (25.23) | 10,090 (26.67) | 9,699 (26.39) |
| NNC | 22,164 (50.66) | 21,751 (51.17) | 17,918 (41.77) | 13,537 (36.67) | 14,108 (39.16) | 12,762 (38.85) | 13,216 (38.25) | 13,379 (38.59) | 14,563 (38.49) | 13,668 (37.18) |

Note: FSM = Full splice match; ISM = incomplete splice match; NIC = novel in catalogue; NNC = novel not in catalogue

| **Table S3** Quality control of RNA-seq data | | | | | | | | |
| --- | --- | --- | --- | --- | --- | --- | --- | --- |
| Sample | Total reads (million) | Base (Gb) | Q30 (%) | GC Content (%) | Uniquely mapped reads (%) | Number of reads mapped to multiple loci (%) | Unmapped (%) | TIN (median) |
| BMI_Tes_1 | 22.41 | 6.72 | 94.16 | 54.60 | 91.06 | 1.90 | 7.04 | 76.68 |
| BMI_Tes_2 | 24.47 | 7.34 | 94.27 | 53.72 | 91.44 | 1.69 | 6.86 | 77.15 |
| BMI_Tes_3 | 33.72 | 10.12 | 93.86 | 54.81 | 91.40 | 1.78 | 6.82 | 75.98 |
| DSE_Tes_1 | 27.91 | 8.37 | 93.84 | 54.47 | 91.22 | 1.93 | 6.85 | 74.94 |
| DSE_Tes_2 | 20.79 | 6.24 | 94.45 | 53.22 | 91.14 | 2.02 | 6.83 | 78.93 |
| DSE_Tes_3 | 26.58 | 7.97 | 94.30 | 54.30 | 91.33 | 1.89 | 6.78 | 77.26 |
| BMI_Epi_1 | 19.32 | 5.80 | 94.10 | 54.39 | 90.41 | 2.14 | 7.45 | 79.62 |
| BMI_Epi_2 | 22.65 | 6.79 | 94.28 | 52.81 | 90.79 | 1.99 | 7.22 | 79.52 |
| BMI_Epi_3 | 19.43 | 5.83 | 94.22 | 51.99 | 91.36 | 1.68 | 6.96 | 76.11 |
| DSE_Epi_1 | 27.26 | 8.18 | 93.47 | 52.76 | 89.66 | 1.74 | 8.60 | 67.68 |
| DSE_Epi_2 | 23.56 | 7.07 | 94.27 | 52.70 | 90.63 | 1.86 | 7.51 | 81.33 |
| DSE_Epi_3 | 25.18 | 7.56 | 94.39 | 52.59 | 90.12 | 2.62 | 7.26 | 80.64 |
| BMI_VG_1 | 24.09 | 7.23 | 95.00 | 51.89 | 90.73 | 3.12 | 6.15 | 75.93 |
| BMI_VG_2 | 23.06 | 6.92 | 94.38 | 52.66 | 90.04 | 3.46 | 6.51 | 79.61 |
| BMI_VG_3 | 19.23 | 5.77 | 94.50 | 52.09 | 89.03 | 4.94 | 6.03 | 78.45 |
| DSE_VG_1 | 25.91 | 7.77 | 94.80 | 51.14 | 89.64 | 4.51 | 5.85 | 73.82 |
| DSE_VG_2 | 21.97 | 6.59 | 94.45 | 51.74 | 89.15 | 4.57 | 6.28 | 78.72 |
| DSE_VG_3 | 22.85 | 6.85 | 94.54 | 51.11 | 89.58 | 4.56 | 5.86 | 78.42 |
| BMI_PG_1 | 36.13 | 10.84 | 95.13 | 53.51 | 92.13 | 1.54 | 6.33 | 79.92 |
| BMI_PG_2 | 27.23 | 8.17 | 95.41 | 52.14 | 90.44 | 3.76 | 5.80 | 79.66 |
| BMI_PG_3 | 23.09 | 6.93 | 94.81 | 51.84 | 91.72 | 1.89 | 6.39 | 73.92 |
| DSE_PG_1 | 28.21 | 8.46 | 94.69 | 54.64 | 92.20 | 1.80 | 6.00 | 79.89 |
| DSE_PG_2 | 24.34 | 7.30 | 93.40 | 52.29 | 92.50 | 1.49 | 6.01 | 80.23 |
| DSE_PG_3 | 25.74 | 7.72 | 94.16 | 53.97 | 91.92 | 1.55 | 6.53 | 80.53 |
| BMI_BG_1 | 26.99 | 8.10 | 94.21 | 54.84 | 90.03 | 2.38 | 7.58 | 76.72 |
| BMI_BG_2 | 28.28 | 8.48 | 95.02 | 53.10 | 91.05 | 2.18 | 6.77 | 80.87 |
| BMI_BG_3 | 26.62 | 7.98 | 94.12 | 54.37 | 90.11 | 2.95 | 6.95 | 80.06 |
| DSE_BG_1 | 22.79 | 6.84 | 94.00 | 54.35 | 89.49 | 3.15 | 7.36 | 79.83 |
| DSE_BG_2 | 22.56 | 6.77 | 94.44 | 52.97 | 91.30 | 2.11 | 6.59 | 73.86 |
| DSE_BG_3 | 23.81 | 7.14 | 94.20 | 54.42 | 89.59 | 3.13 | 7.28 | 80.24 |

**Table S4** Quality control of small RNA-seq data

| Sample | Total reads (million) | Base (Gb) | length (mean) | Q30 (%) | GC content (%) | >18 nt reads (million) | Mapped (%) | Unmapped (%) |
| --- | --- | --- | --- | --- | --- | --- | --- | --- |
| BMI_BG_1 | 18.21 | 0.43 | 23.60 | 89.33 | 44.35 | 17.93 | 85.37 | 14.63 |
| BMI_BG_2 | 20.37 | 0.46 | 22.30 | 90.92 | 45.09 | 19.21 | 90.03 | 9.98 |
| BMI_BG_3 | 21.32 | 0.50 | 23.50 | 90.31 | 44.37 | 20.79 | 84.00 | 16.00 |
| BMI_Epi_1 | 20.34 | 0.49 | 24.20 | 89.29 | 46.59 | 20.27 | 82.68 | 17.32 |
| BMI_Epi_2 | 19.45 | 0.45 | 23.10 | 89.81 | 45.41 | 18.97 | 87.88 | 12.12 |
| BMI_Epi_3 | 22.12 | 0.48 | 21.80 | 90.94 | 45.01 | 21.53 | 92.62 | 7.38 |
| BMI_PG_1 | 19.67 | 0.45 | 22.80 | 89.32 | 44.89 | 19.50 | 89.42 | 10.58 |
| BMI_PG_2 | 16.80 | 0.37 | 22.10 | 90.14 | 45.06 | 16.30 | 92.14 | 7.86 |
| BMI_PG_3 | 17.50 | 0.40 | 22.90 | 91.21 | 43.12 | 17.38 | 89.13 | 10.87 |
| BMI_Tes_1 | 21.08 | 0.61 | 29.10 | 88.99 | 45.05 | 20.98 | 81.86 | 18.14 |
| BMI_Tes_2 | 24.40 | 0.69 | 28.20 | 89.27 | 45.40 | 24.05 | 81.84 | 18.16 |
| BMI_Tes_3 | 20.59 | 0.57 | 27.70 | 88.96 | 45.60 | 20.20 | 81.46 | 18.55 |
| BMI_VG_1 | 17.38 | 0.40 | 22.90 | 90.73 | 42.89 | 17.29 | 87.62 | 12.38 |
| BMI_VG_2 | 17.15 | 0.39 | 22.60 | 89.45 | 44.80 | 16.89 | 89.70 | 10.30 |
| BMI_VG_3 | 19.75 | 0.44 | 22.40 | 90.56 | 44.40 | 19.24 | 90.81 | 9.19 |
| DSE_BG_1 | 20.80 | 0.45 | 21.70 | 91.25 | 45.24 | 18.69 | 92.60 | 7.40 |
| DSE_BG_2 | 21.06 | 0.47 | 22.50 | 89.90 | 44.50 | 20.23 | 91.23 | 8.77 |
| DSE_BG_3 | 22.22 | 0.52 | 23.20 | 91.10 | 43.29 | 21.89 | 87.49 | 12.51 |
| DSE_Epi_1 | 22.39 | 0.50 | 22.20 | 90.25 | 44.80 | 22.00 | 92.43 | 7.57 |
| DSE_Epi_2 | 23.14 | 0.50 | 21.50 | 89.86 | 46.87 | 22.88 | 91.80 | 8.20 |
| DSE_Epi_3 | 23.56 | 0.51 | 21.70 | 90.41 | 46.75 | 22.79 | 91.49 | 8.51 |
| DSE_PG_1 | 24.10 | 0.53 | 21.80 | 89.71 | 45.50 | 23.66 | 92.35 | 7.65 |
| DSE_PG_2 | 20.48 | 0.46 | 22.30 | 90.07 | 45.94 | 19.51 | 90.22 | 9.78 |
| DSE_PG_3 | 14.76 | 0.32 | 21.70 | 89.90 | 45.74 | 14.30 | 92.89 | 7.11 |
| DSE_Tes_1 | 21.39 | 0.60 | 28.00 | 90.54 | 45.91 | 21.07 | 84.30 | 15.70 |
| DSE_Tes_2 | 21.37 | 0.47 | 22.00 | 89.78 | 46.84 | 18.61 | 87.57 | 12.43 |
| DSE_Tes_3 | 21.40 | 0.51 | 23.70 | 89.78 | 47.53 | 19.10 | 86.58 | 13.42 |
| DSE_VG_1 | 22.13 | 0.49 | 22.10 | 91.89 | 44.23 | 21.70 | 94.15 | 5.85 |
| DSE_VG_2 | 24.93 | 0.54 | 21.80 | 90.34 | 45.36 | 23.77 | 90.82 | 9.18 |
| DSE_VG_3 | 22.51 | 0.49 | 21.90 | 90.93 | 44.37 | 21.82 | 93.88 | 6.12 |
